# Supplementary material for: The interactome of intact mitochondria by cross-linking mass spectrometry provides evidence for coexisting respiratory supercomplexes
Source: Mol Cell Proteomics. 2017 Dec 8;17(2):216–32. doi: 10.1074/mcp.RA117.000470 (PMC5795388; doi:10.1074/mcp.RA117.000470)
Supplement: Supplemental Data [file supp_RA117.000470_133922_0_supp_23978_fzffwf.zip › spectra_annotation/mito_spectra_annotation/101-1-3-1-13-1.pdf]

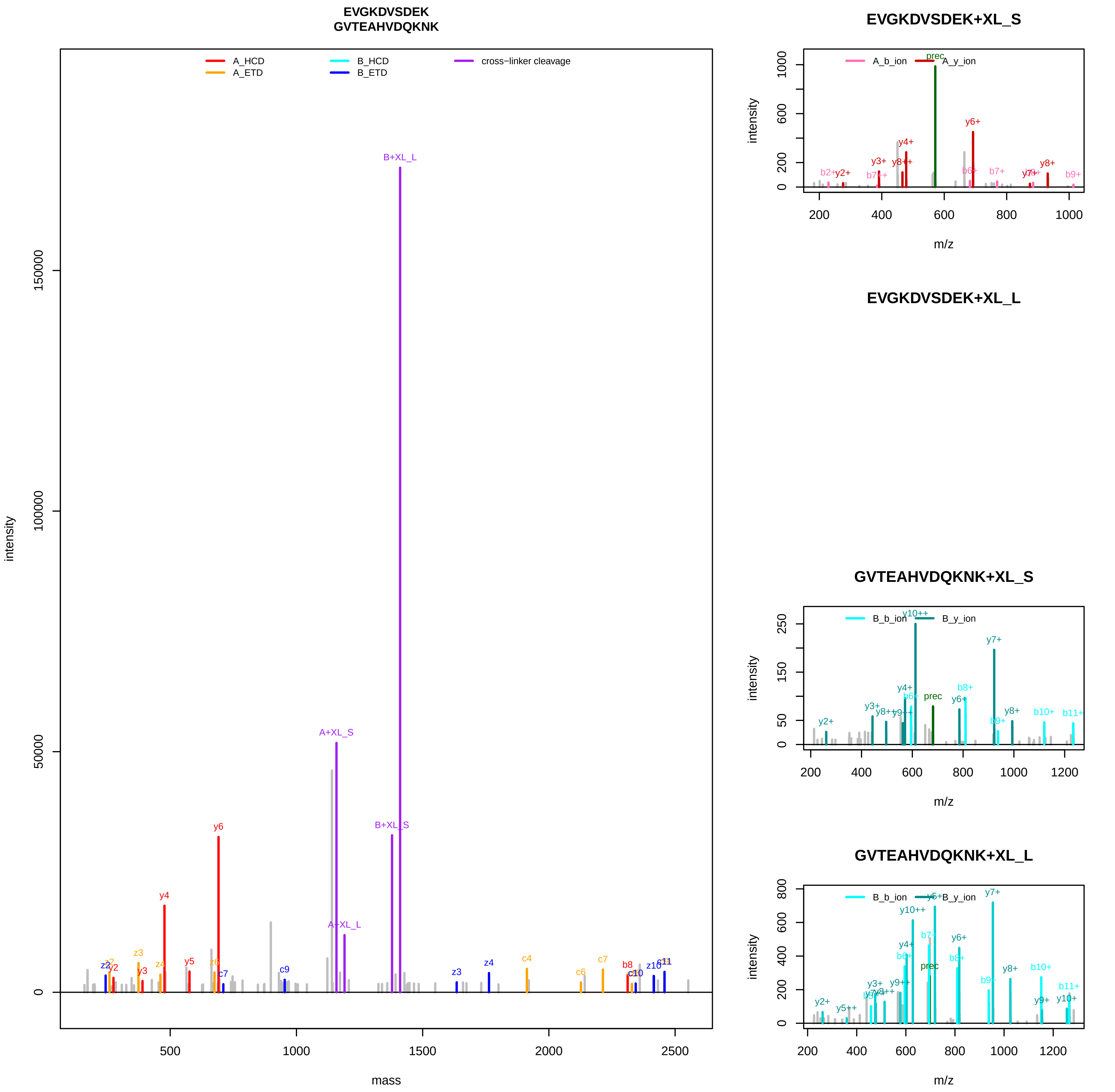
EVGKDVSDEK
GVTEAHVDQKNK
A_HCD
A_ETD
B_HCD
B_ETD
cross−linker cleavage
B+XL_L
A+XL_S
B+XL_S
A+XL_L
intensity
mass
0
50000
100000
150000
500
1000
1500
2000
2500
y4
y5
y6
y2
y3
z2
z3
z4
z6
c7
c9
z3
z4
c4
c6
c7
b8
c10
z10
c11
EVGKDVSDEK+XL_S
A_b_ion
A_y_ion
prec
y6+
y4+
y3+
y8++
b2+
y2+
b7++
b6+
b7+
y7+
y8+
b9+
m/z
EVGKDVSDEK+XL_L
GVTEAHVDQKNK+XL_S
B_b_ion
B_y_ion
y10++
y7+
y4+
b6+
prec
b8+
y6+
y3+
y8++
y9++
y2+
b9+
y8+
b10+
b11+
GVTEAHVDQKNK+XL_L
y5+
y10++
b7+
y6+
y4+
b6+
prec
b8+
y3+
y9++
y5++
b9+
y8+
b10+
b11+
y9+
y10+
